# Hemodynamics and gas exchange during chest compressions in neonatal resuscitation

**DOI:** 10.1371/journal.pone.0176478

**Published:** 2017-04-25

**Authors:** Payam Vali, Praveen Chandrasekharan, Munmun Rawat, Sylvia Gugino, Carmon Koenigsknecht, Justin Helman, Bobby Mathew, Sara Berkelhamer, Jayasree Nair, Myra Wyckoff, Satyan Lakshminrusimha

**Affiliations:** 1 Pediatrics, UC Davis, Sacramento, California, United States of America; 2 Pediatrics, SUNY University at Buffalo, Buffalo, New York, United States of America; 3 Pediatrics, UT Southwestern, Dallas, Texas, United States of America; Centre Hospitalier Universitaire Vaudois, FRANCE

## Abstract

**Purpose:**

Current knowledge about pulmonary/systemic hemodynamics and gas exchange during neonatal resuscitation in a model of transitioning fetal circulation with fetal shunts and fluid-filled alveoli is limited. Using a fetal lamb asphyxia model, we sought to determine whether hemodynamic or gas-exchange parameters predicted successful return of spontaneous circulation (ROSC).

**Methods:**

The umbilical cord was occluded in 22 lambs to induce asphyxial cardiac arrest. Following five minutes of asystole, resuscitation as per AHA-Neonatal Resuscitation Program guidelines was initiated. Hemodynamic parameters and serial arterial blood gases were assessed during resuscitation.

**Results:**

ROSC occurred in 18 lambs (82%) at a median (IQR) time of 120 (105–180) seconds. There were no differences in hemodynamic parameters at baseline and at any given time point during resuscitation between the lambs that achieved ROSC and those that did not. Blood gases at arrest prior to resuscitation were comparable between groups. However, lambs that achieved ROSC had lower PaO_2_, higher PaCO_2_, and lower lactate during resuscitation. Increase in diastolic blood pressures induced by epinephrine in lambs that achieved ROSC (11 ±4 mmHg) did not differ from those that were not resuscitated (10 ±6 mmHg). Low diastolic blood pressures were adequate to achieve ROSC.

**Conclusions:**

Hemodynamic parameters in a neonatal lamb asphyxia model with transitioning circulation did not predict success of ROSC. Lactic acidosis, higher PaO_2_ and lower PaCO_2_ observed in the lambs that did not achieve ROSC may represent a state of inadequate tissue perfusion and/or mitochondrial dysfunction.

## Introduction

The need for chest compressions is infrequent in neonatal resuscitation with an estimated occurrence of 0.08% for near-term and term deliveries, and a higher frequency (2–10%) in preterm infants.[1] Among the survivors who require chest compressions at birth, many suffer from long-term neurologic deficits.[2, 3] Therefore, in the event of circulatory collapse in the severely asphyxiated newborn infant, swiftly establishing return of spontaneous circulation (ROSC) is paramount to prevent any long-term detrimental neurologic sequelae. The success in achieving ROSC without neurologic deficit lies primarily in efficient restoration of gas exchange with ventilation and oxygenation, as well as provision of adequate blood supply to the coronary and cerebral circulation through chest compressions. Integral to sustaining optimal circulation is achieving adequate gas exchange not only at the level of the lungs but also in the tissues where oxygen utilization depends on mitochondrial integrity and adequate cellular function.

The scientific evidence behind the current recommendations for chest compressions during neonatal resuscitation remains limited: conclusions are extrapolated from animal, pediatric, and adult literature, as well as physiologic plausibility and expert opinion. Few studies have reported on hemodynamic parameters during neonatal resuscitation,[4–9] and there is even less data depicting the transitioning fetal circulation in the context of fluid-filled alveoli and a patent ductus arteriosus (PDA).[7]

Nevertheless, hemodynamic-directed cardiopulmonary resuscitation (CPR) in a piglet model of asphyxia-associated cardiac arrest has demonstrated improved survival.[10] We hypothesize that hemodynamics and blood gas parameters during neonatal CPR influence success in achieving ROSC. Hemodynamic data and arterial blood gas (ABG) analysis collected during resuscitation in a term lamb model of asphyxia-induced cardiac arrest with transitioning fetal circulation were compiled and analyzed.

## Methods

### Animal preparation

**Fig 1:**
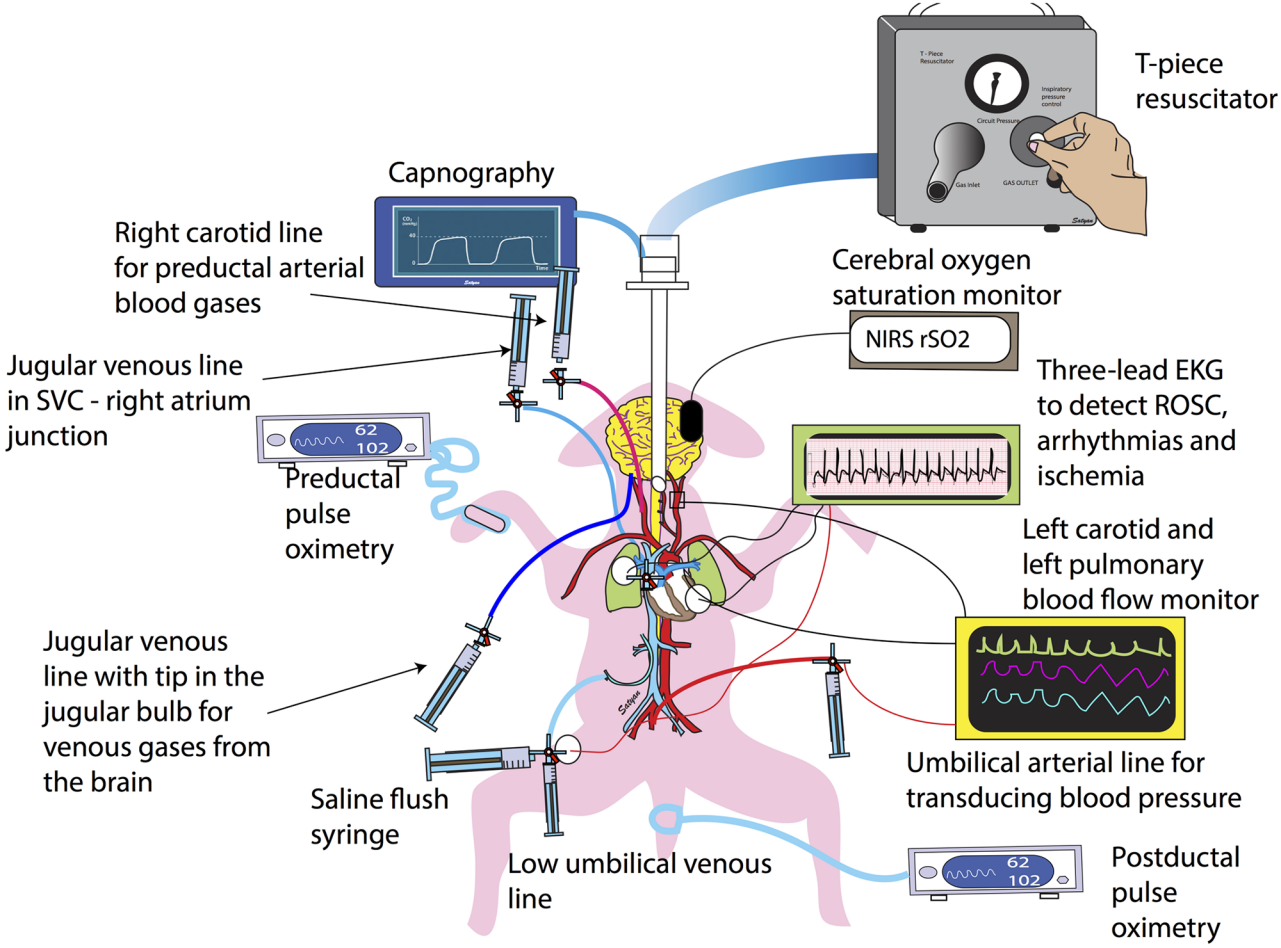
Illustration of methodology depicting invasive and non-invasive monitoring.

This study was approved by the Institutional Animal Care and Use Committee at the State University of New York at Buffalo. Time-dated term (139–141 day gestation) pregnant ewes were obtained from New Pasteur Family Farms (Attica, NY). Following an overnight fast, the ewes were induced for anesthesia with intravenous diazepam and ketamine. They were intubated with a 10.0-mm cuffed endotracheal tube (ETT) and ventilated with 21% oxygen and 2–3% isoflurane at 16 breaths/min. The ewes were continuously monitored with a pulse oximeter and an end-tidal CO_2_ (EtCO_2_) monitor. Following cesarean section, fetal lambs were partially exteriorized and intubated with a 4.5-mm cuffed ETT. The fetal lung fluid in the ETT was drained passively by gravity by tilting the head to the side and, thereafter, the ETT was occluded to prevent gas exchange during gasping. Catheters were inserted into the right jugular vein (for fluid and medication administration) and right carotid artery (fetal blood pressure monitoring). A 2 mm flow probe (Transonic Systems Inc, Ithaca NY) was placed around the left carotid artery. A left thoracotomy was performed and a 4 mm flow probe was placed around the left pulmonary artery. The thoracotomy was closed in layers. Preductal pulse oximetry (SpO_2_) and regional cerebral tissue oxygen saturation (CrSO_2_) were monitored using NONIN Equanox (Nonin Medical Inc, Plymouth, MN). After cutting the umbilical cord, the umbilical artery was catheterized for neonatal blood pressure monitoring (Fig 1).

### Experimental protocol

After instrumentation, the lamb was delivered onto a radiant warmer. Asystole was defined by the absence of carotid blood flow, arterial blood pressure and heart rate (auscultation and EKG). As part of a feasibility trial, in an attempt to resuscitate lambs with asphyxia-induced cardiac arrest, we learned that the lambs readily achieved ROSC with positive pressure ventilation (PPV) without requiring chest compressions when resuscitation was initiated immediately after asystole. Therefore, a five-minute period of asystole was observed prior to initiating resuscitation. One minute prior to resuscitation (four minutes into asystole), the first arterial blood sample was obtained (“arrest gas”) and, thereafter, blood sampling was obtained every minute during resuscitation. In lambs that achieved ROSC, a blood gas sample was obtained at the time of ROSC and then every minute until 10 minutes following ROSC. Arterial blood samples were immediately analyzed using a radiometer blood gas analyzer (ABL 800 FLEX, Denmark).

Following five minutes of asystole, resuscitation began per the Neonatal Resucitation Program (NRP) guidelines.[11] The ETT occluder was removed and PPV was provided by means of a T-piece resuscitator at pressures of 35/5 cm H_2_O at a rate of 40 breaths/min with an FIO_2_ of 0.21 that was increased to 1.0 before initiation of chest compressions. Pressures of 35/5 cm H_2_O are required to achieve tidal volumes of approximately 8–9 ml/kg in term lambs, which are necessary to achieve normal PaCO_2_ levels. Following ROSC, the PIP was weaned gradually based on tidal volume and PaCO_2_. Following 30 seconds of ventilation through the endotracheal tube, chest compressions at a compression to ventilation (C:V) ratio of 3:1 was commenced if ROSC was not achieved. Chest compressions were provided by a single resuscitator (SL) and coordinated with PPV while counting out loud “one-and-two-and-three and breathe, and…” as recommended by NRP. The first dose of epinephrine (0.03 mg/kg) was given intravenously 30 seconds following initiation of chest compressions, then every 3 minutes until ROSC (maximum of 4 doses) were administered. If ROSC was achieved, the FIO_2_ was gradually weaned to maintain a target preductal SpO2 of 85–95%.

### Data analysis

Arterial blood flows and pressures were continuously recorded using a computer with AcqKnowledge Acquisition & Analysis Software (BIOPAC systems, Goleta, CA, USA). Continuous variables are expressed as mean and standard deviation. Categorical variables were analyzed using chi square test with Fisher’s exact test as required. Continuous variables were analyzed by unpaired t-test. A probability <5% was considered statistically significant.

### Results

#### Lamb characteristics and ROSC

A total of 22 lambs were studied. The time to instrument the lambs was approximately 65 minutes and there was no difference between the groups. The first lamb of multiple gestation pregnancies was exposed to approximately 75 minutes of maternal anesthesia, while the second twin or triplet was exposed to a 140 minutes. In the “ROSC” group, there were 9/18 singleton and first-oder lambs compared to 3/4 lambs in the “no ROSC” group. A significant greater number of lambs in the “ROSC” group (9/18) were exposed to longer maternal anesthesia compared to the “no ROSC” group (1/4). Lamb characteristics, hemodynamic parameters and arrest ABG analysis were similar between the groups (Tables 1 and 2). 18/22 lambs (82%) achieved ROSC in a median time (IQR) of 120 (105–180) seconds. Hemodynamic parameters and blood gas analysis following ROSC are presented in Tables 1 and 2.

**Table 1:** Lamb characteristics and hemodynamic parameters.

	ROSC (n = 18)	No ROSC (n = 4)
Weight (Kg)	3.5 ± 0.8	3.8 ± 0.3
Sex (M:F)	10:8	1:3
Singleton or First-of-multiples Lambs	9	1
Total lung fluid drained (ml)	14 ± 3.2	12 ± 4.4
**Baseline Hemodynamics**		
Heart Rate (bpm)	159 ± 25	177 ± 15
SBP mean (mm Hg)	58 ± 10	55 ± 15
DBP mean (mm Hg)	42 ± 8	37 ± 14
Mean left carotid flow (ml/kg/min)	25 ± 10	26 ± 5
Mean left PA flow (ml/kg/min)	28 ± 21	21 ± 14
**Hemodynamics during Resuscitation**		
CC Prior to Epinephrine		
Compressions/min	88 ± 9	93 ± 6
SBP mean (mm Hg)	26 ± 10	25 ± 8
DBP mean (mm Hg)	8 ± 4	5 ± 3
Mean left carotid flow (ml/kg/min)	4 ± 2	2 ± 2
Mean left PA flow (ml/kg/min)	2± 4	1± 2
CC Following Epinephrine		
CC rate (bpm)	86 ± 9	92 ± 10
SBP mean (mm Hg)	32 ± 9	33 ± 13
DBP mean (mm Hg)	11 ± 4	10 ± 6
Mean left carotid flow (ml/kg/min)	4 ± 3	3 ± 2
Mean left PA flow (ml/kg/min)	3 ± 3	3 ± 6
10 min After ROSC		
Heart rate (bpm)	204 ± 17	
SBP mean (mm Hg)	82 ± 19	
DBP mean (mm Hg)	59 ± 17	
Mean left carotid flow (ml/kg/min)	28 ± 10	
Mean left PA flow (ml/kg/min)	94 ± 86	

Baseline characteristics and hemodynamic parameters during chest compressions (CC) prior to epinephrine, following epinephrine and 10 minutes following return of spontaneous circulation (ROSC). Mean values ± SD are shown. PA: pulmonary artery.

**Table 2:** Arterial blood gas analysis.

	ROSC N = (18)	No ROSC (n = 4)
**At Arrest**		
Arterial pH	6.89 ± 0.03	6.86 ± 0.08
PaCO_2_ (mmHg)	146 ± 24	109 ± 33*
PaO_2_ (mmHg)	5 ± 3	7 ± 4
Lactate (mmol/L)	12 ± 3	14 ± 6
	**Last ABG prior to ROSC**	
Arterial pH	6.84 ± 0.08	
PaCO_2_ (mm Hg)	131 ± 21	
PaO_2_ (mm Hg)	19 ± 6.5	
Lactate (mmol/L)	13 ± 2	
	**10 Min Following ROSC**	**15 Min Post Arrest**
Arterial pH	7.1 ± 0.14	6.8 ± 0.1
PaCO_2_ (mm Hg)	61 ± 24	67 ± 14
PaO_2_ (mm Hg)	59 ± 30^¶^	36 ± 5^†^
Lactate (mmol/L)	11 ± 4	16 ± 3

Mean values ± SD are shown.

* *P* <0.05 compared to the return of spontaneous circulation (ROSC) group.

^¶^ on 21–85% inspired oxygen;

^†^ on 100% inspired oxygen.

#### Hemodynamic parameters during the initiation of chest compressions

**Fig 2:**
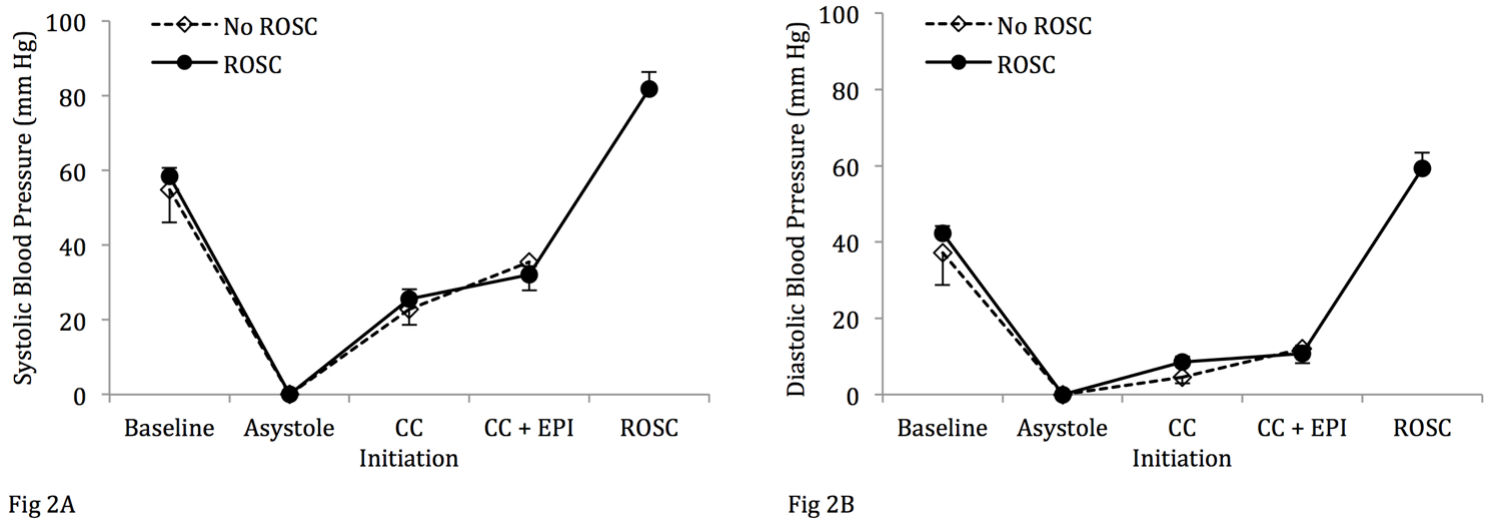
Arterial blood pressures during study period. There is no difference in mean systolic blood pressures (A) and mean diastolic blood pressures (B) between lambs that achieve return of spontaneous circulation (ROSC) and those with no ROSC. Epinephrine did not statistically increase blood pressures in either group. Data are mean ± SEM bars.

**Fig 3:**
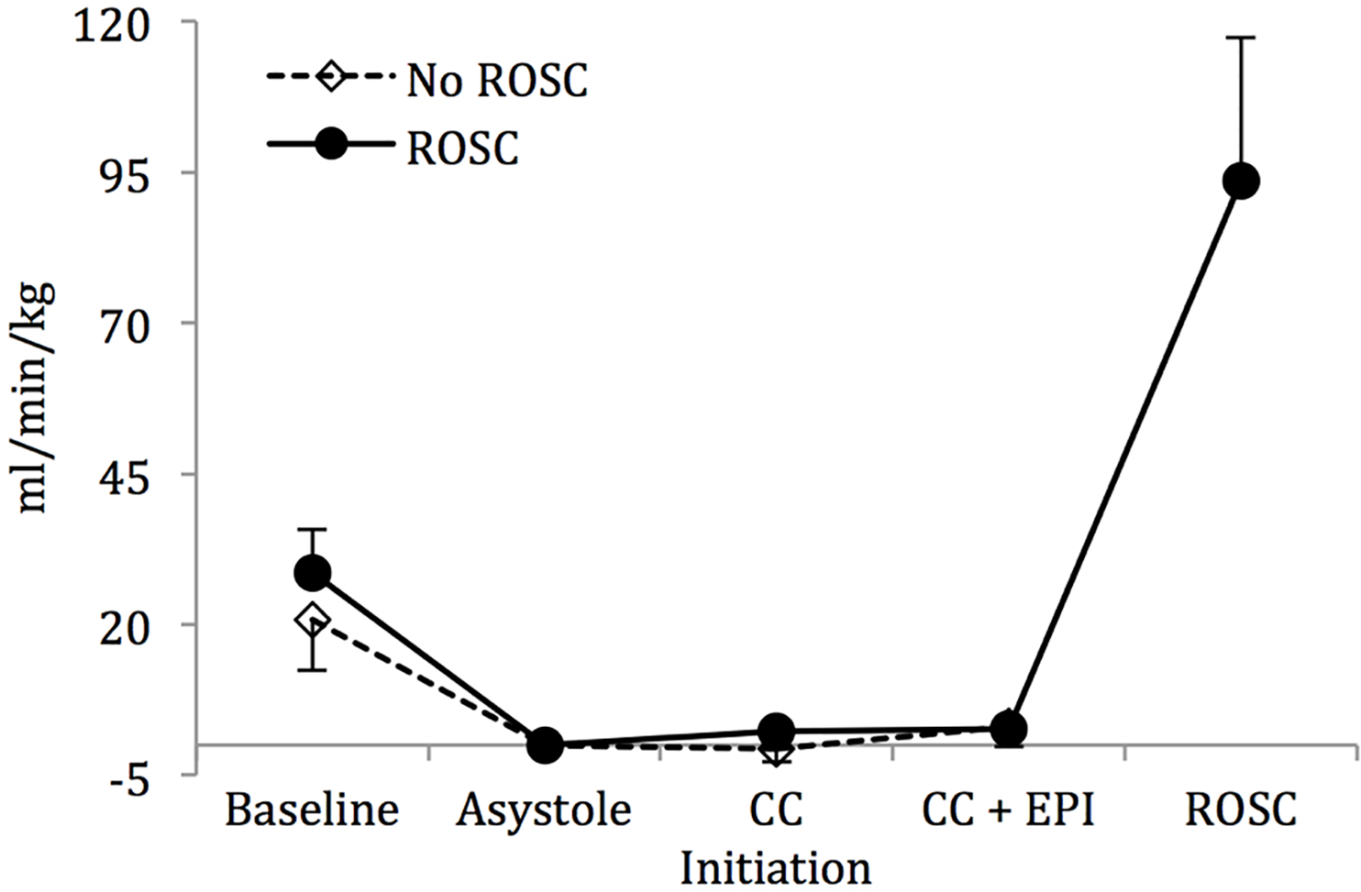
Mean left pulmonary artery flow. There is no appreciable increase in flow following initiation of chest compressions or after epinephrine administration. Data are mean ± SEM bars.

**Fig 4:**
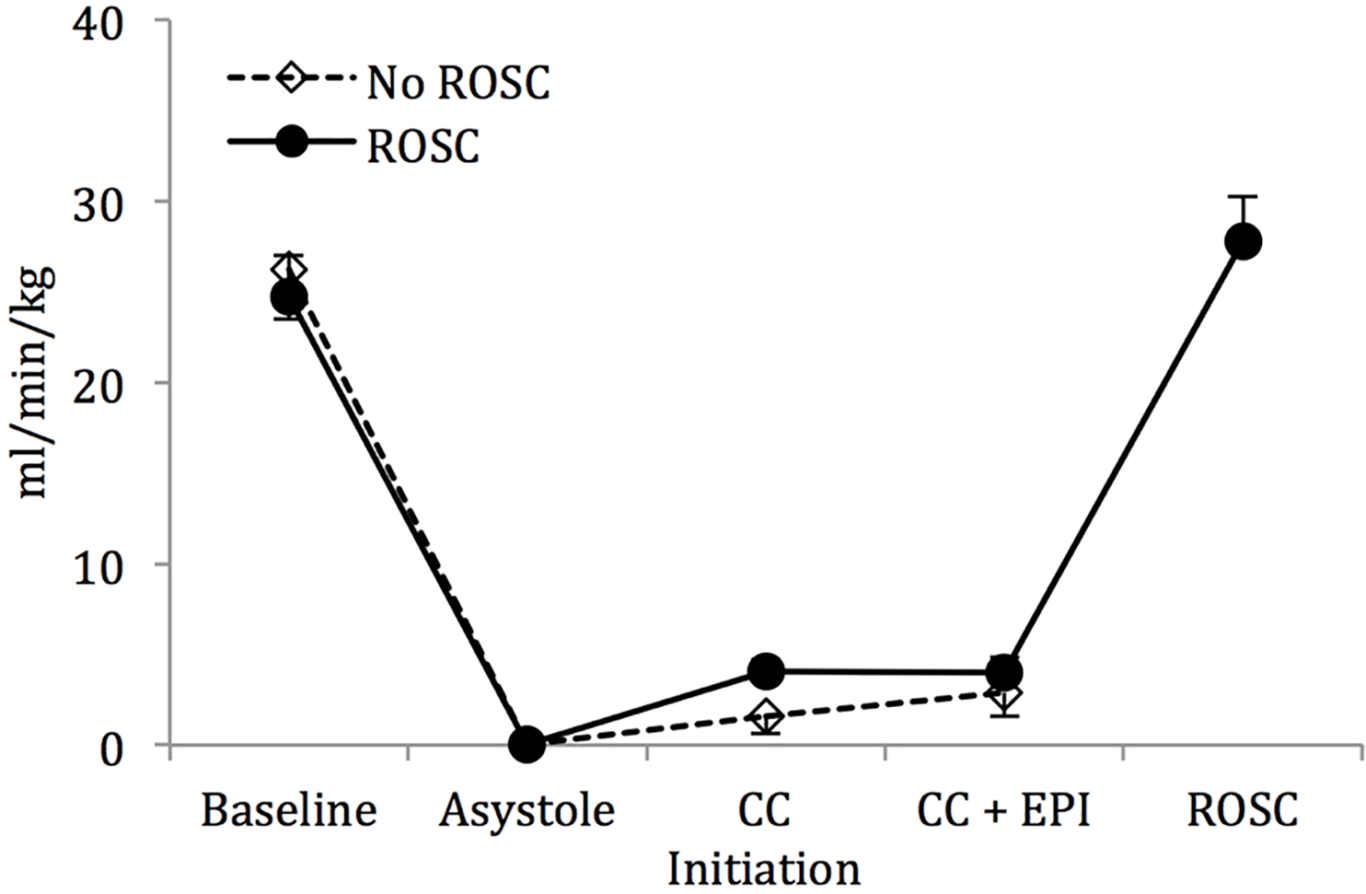
Mean left carotid artery flow. There is only a modest increase in flow following initiation of chest compressions with no further increase after epinephrine use. Data are mean ± SEM bars.

**Fig 5:**
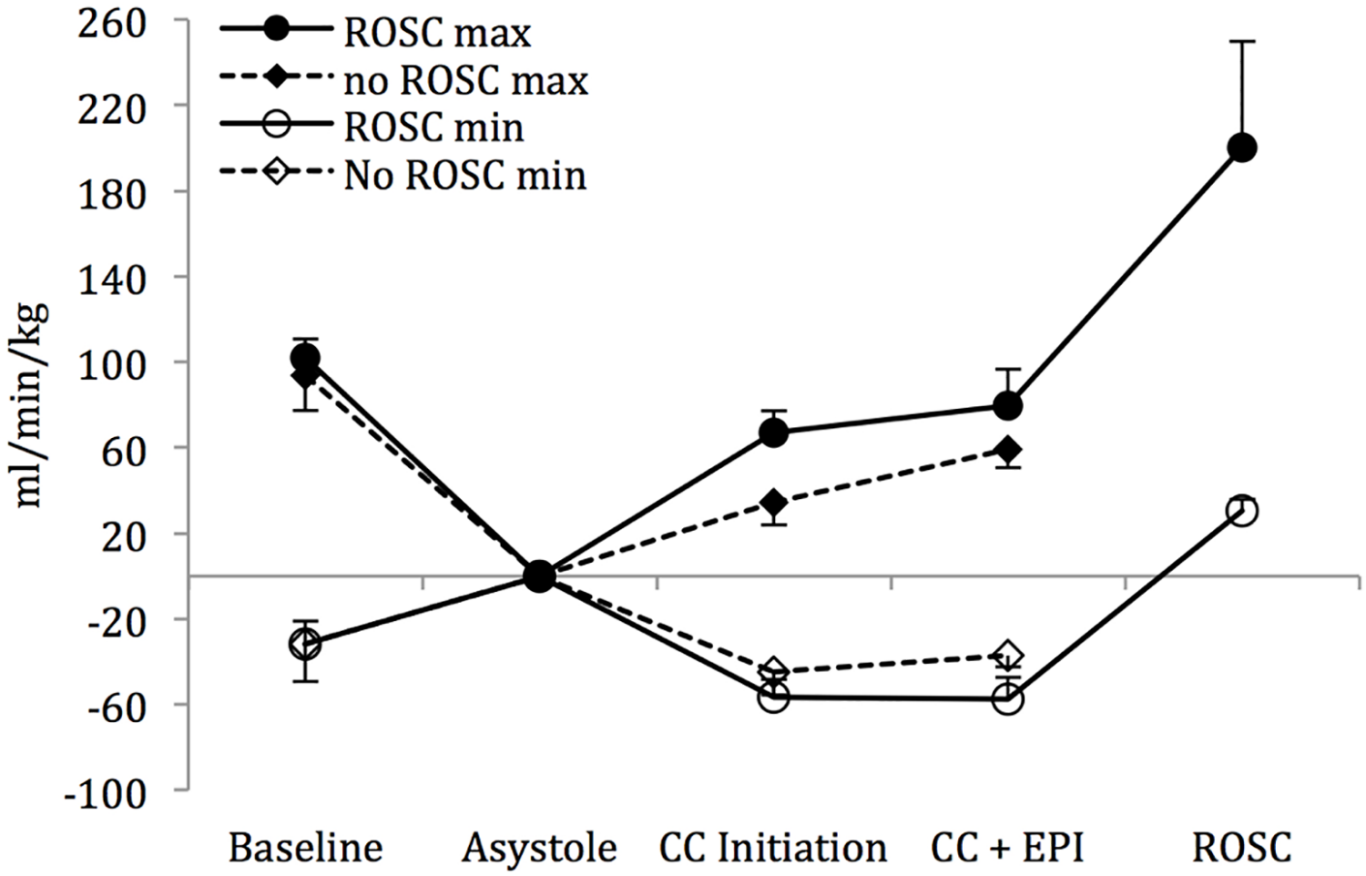
Left pulmonary artery flow. Maximum flow increases with initiation of chest compressions in lambs with ROSC and no ROSC. Reversal of flow occurs in diastole (minimum flow) at baseline (in utero), and remains retrograde during the relaxation phase of chest compressions in lambs of both groups. Flow becomes exclusively anterograde upon ROSC and cessation of chest compressions. Data are mean ± SEM bars.

**Fig 6:**
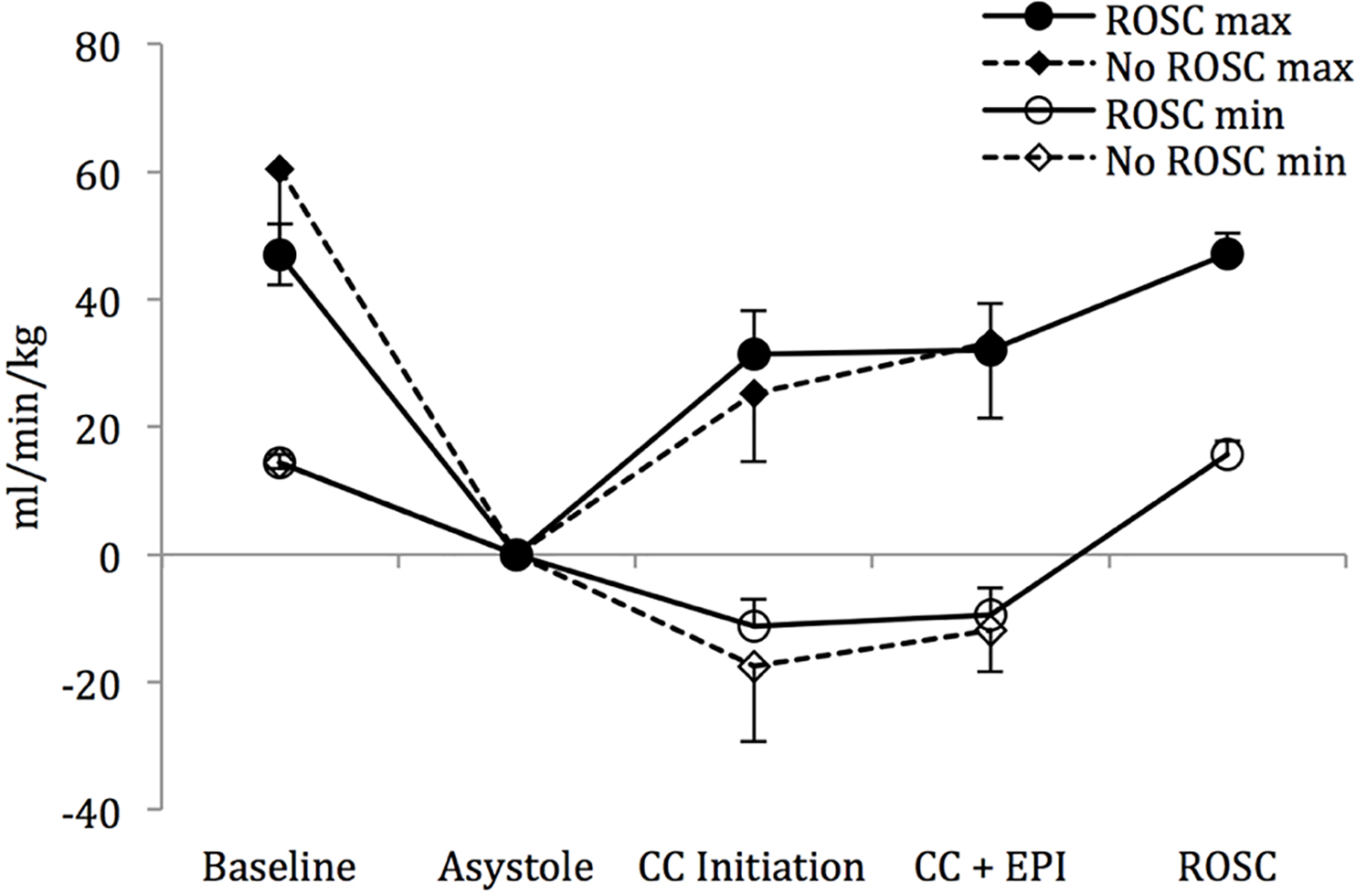
Left carotid artery flow. Maximum left carotid flow increases with initiation of chest compressions in lambs with ROSC and no ROSC. During the relaxation phase of chest compressions (minimum flow), retrograde flow is observed in lambs of both groups. Flow becomes exclusively anterograde upon ROSC and cessation of chest compressions. Data are mean ± SEM bars.

Mean systolic (Fig 2A) and diastolic (Fig 2B) blood pressures increased upon chest compression initiation but did not differ between the lambs that were successfully resuscitated to those that did not achieve ROSC. There was no appreciable increase in the mean left pulmonary flows following chest compressions in either group (Fig 3), while there was only a modest rise in the mean left carotid artery flows (Fig 4). However, there was an appreciable increase in the maximum left pulmonary (Fig 5) and carotid artery (Fig 6) flows during the compression phase of the chest compressions. Interestingly, retrograde flow was observed during the recoil (relaxation) phase of the chest compression cycle. Upon ROSC, with cessation of chest compressions, anterograde flow through the left pulmonary artery and left carotid artery was maintained during systole and diastole (Figs 5 and 6).

#### Effects of chest compressions and epinephrine on hemodynamic parameters

All lambs required epinephrine to achieve ROSC. Sixteen lambs required one dose of epinephrine and 2/18 lambs that were successfully resuscitated required multiple doses of epinephrine (≥ 2). Following the administration of epinephrine during chest compressions in the lambs that achieved ROSC, the mean systolic and diastolic blood pressures rose from 26 ±10 / 8 ±4 to 32 ±9 / 11 ±4 mm Hg, which did not meet statistical significance (Table 1 and Fig 2). Similarly, in the lambs that did not achieve ROSC, epinephrine had a modest effect on the mean systolic / diastolic pressures from 25 ±8 / 5 ±3 to 33 ±13 / 10 ±6 mm Hg that did not reach statistical significance. Epinephrine also did not have a significant effect on carotid and pulmonary arterial flows (Table 1, Figs 5 and 6).

#### Arterial blood gas analysis

**Fig 7:**
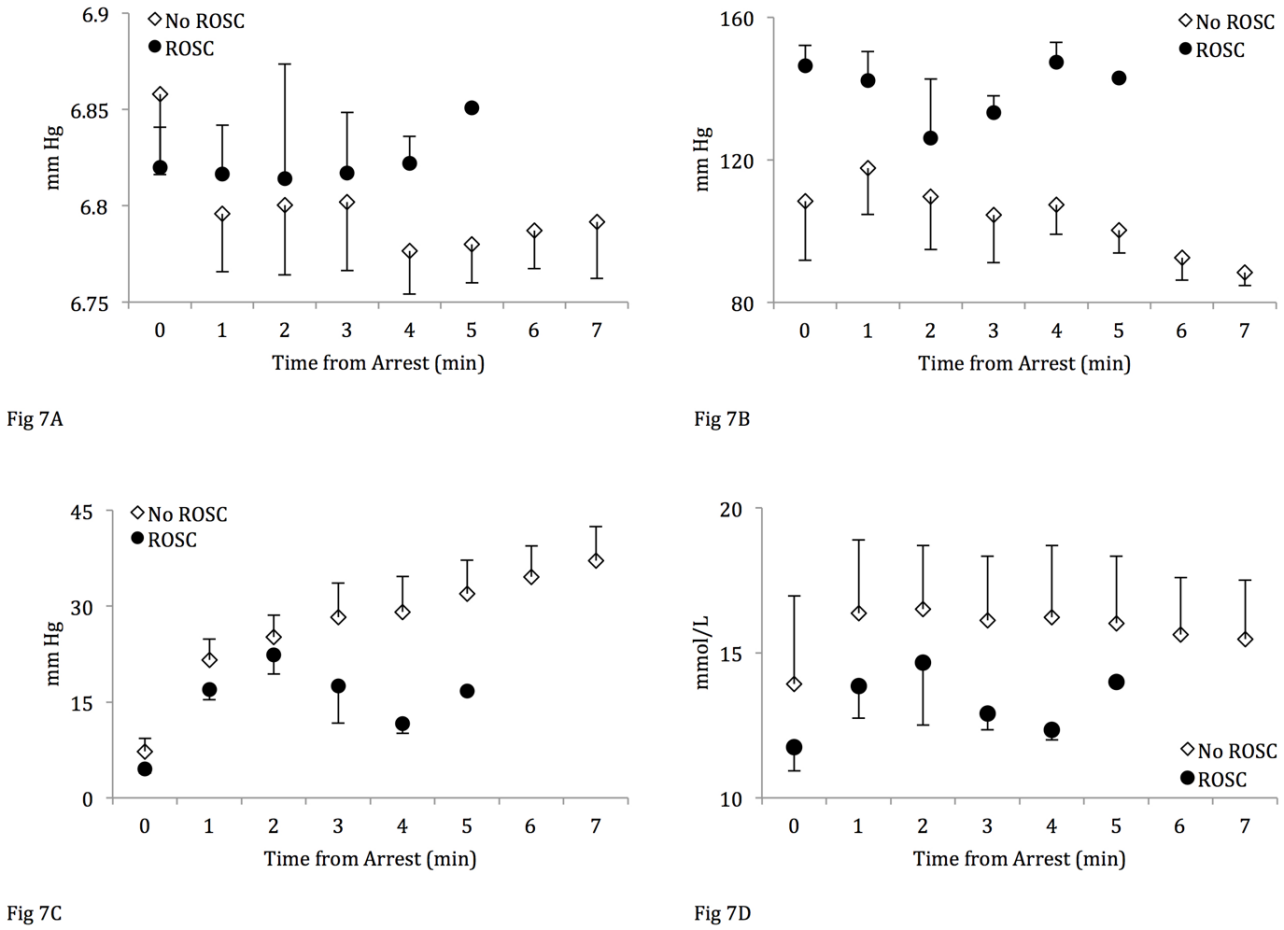
Comparison of arterial blood gas analysis between groups during resuscitation. (A) PaO_2_, (B) PaCO_2_, (C) pH, (D) Lactate. Gas sample “0” depicts “arrest gas.” Data are mean ± SEM bars. The number of lambs in the ROSC group drops with each successful ROSC (at “0” n = 18, at “1” n = 17, at “2” n = 8, at “3” n = 4, at “4” n = 2, at “6” n = 1).

**Fig 8:**
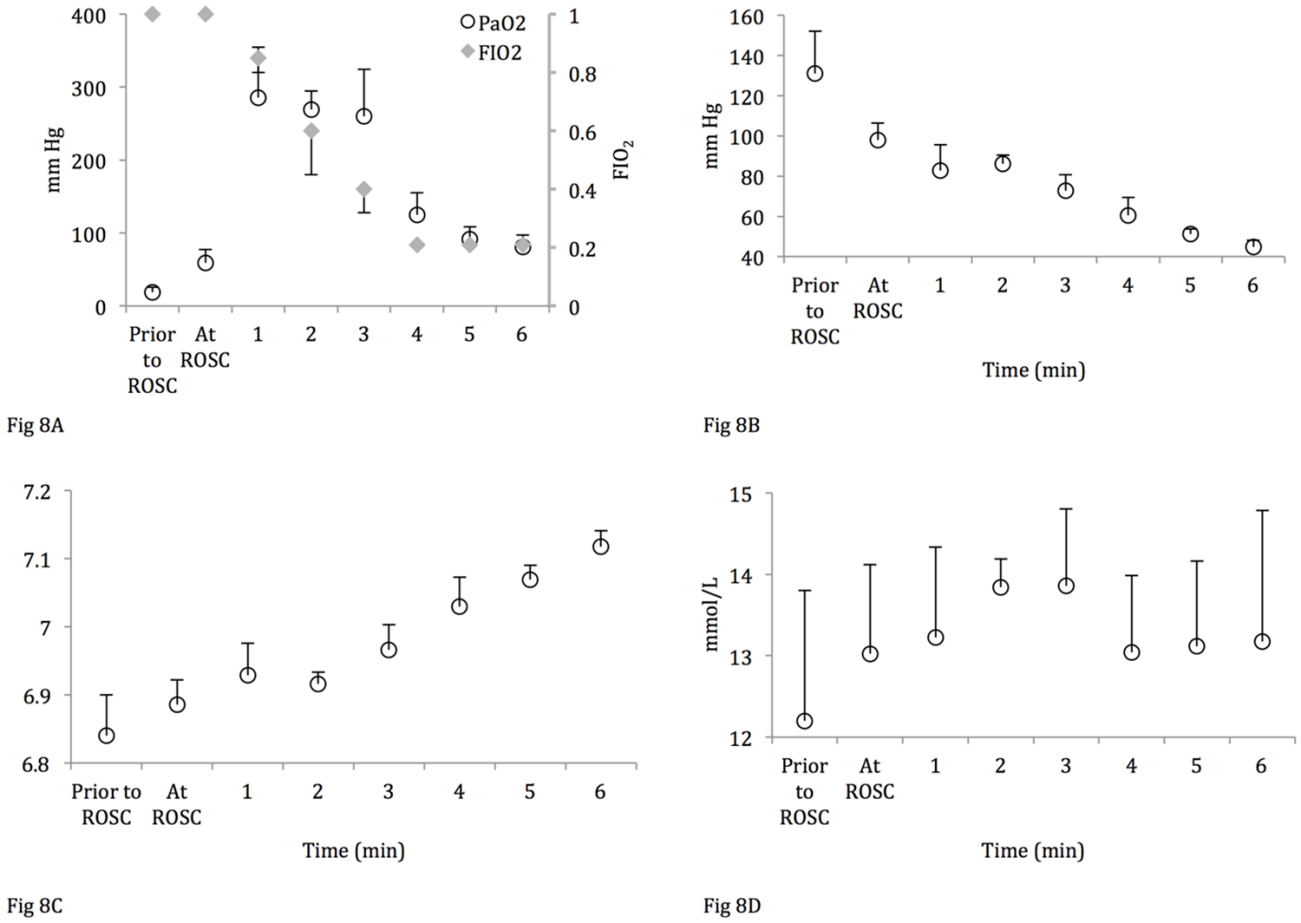
Comparison of arterial blood gas analysis following ROSC. (A) PaO_2_; the secondary y-axis represents FIO_2_, (B) PaCO_2_, (C) pH, (D) Lactate. Data are mean ± SEM bars.

Blood gas samples collected at “arrest,” defined as four minutes from the time of asystole (one minute prior to the onset of resuscitation), showed a lower PaCO_2_ in the lambs that did not achieve ROSC (Table 2). Lambs that achieved ROSC had overall lower PaO_2_, higher PaCO_2_ and lower lactate values during resuscitation compared to lambs that did not achieve ROSC (Fig 7A–7D). The ABG values 10 minutes after ROSC and fifteen minutes into resuscitation (lambs with no ROSC) are shown in Table 2. Despite providing PPV with 100% oxygen and effective ventilation, mean PaO_2_ levels remained low prior to ROSC at 18 ±7 mm Hg (oxygen hemoglobin saturation of ~50%). In the setting of a mean hemoglobin concentration of 12 ±2 g/dL, the mean arterial oxygen content during resuscitation was only 8 ±2 mL O_2_/dL. Following ROSC, PaO_2_ rapidly increased enabling the resuscitators to rapidly wean inspired oxygen (Fig 8A). ROSC resulted in a rapid decrease in PaCO_2_ associated with improvement in pH (Fig 8B and 8C) with no significant change in lactate (Fig 8D).

**Fig 9:**
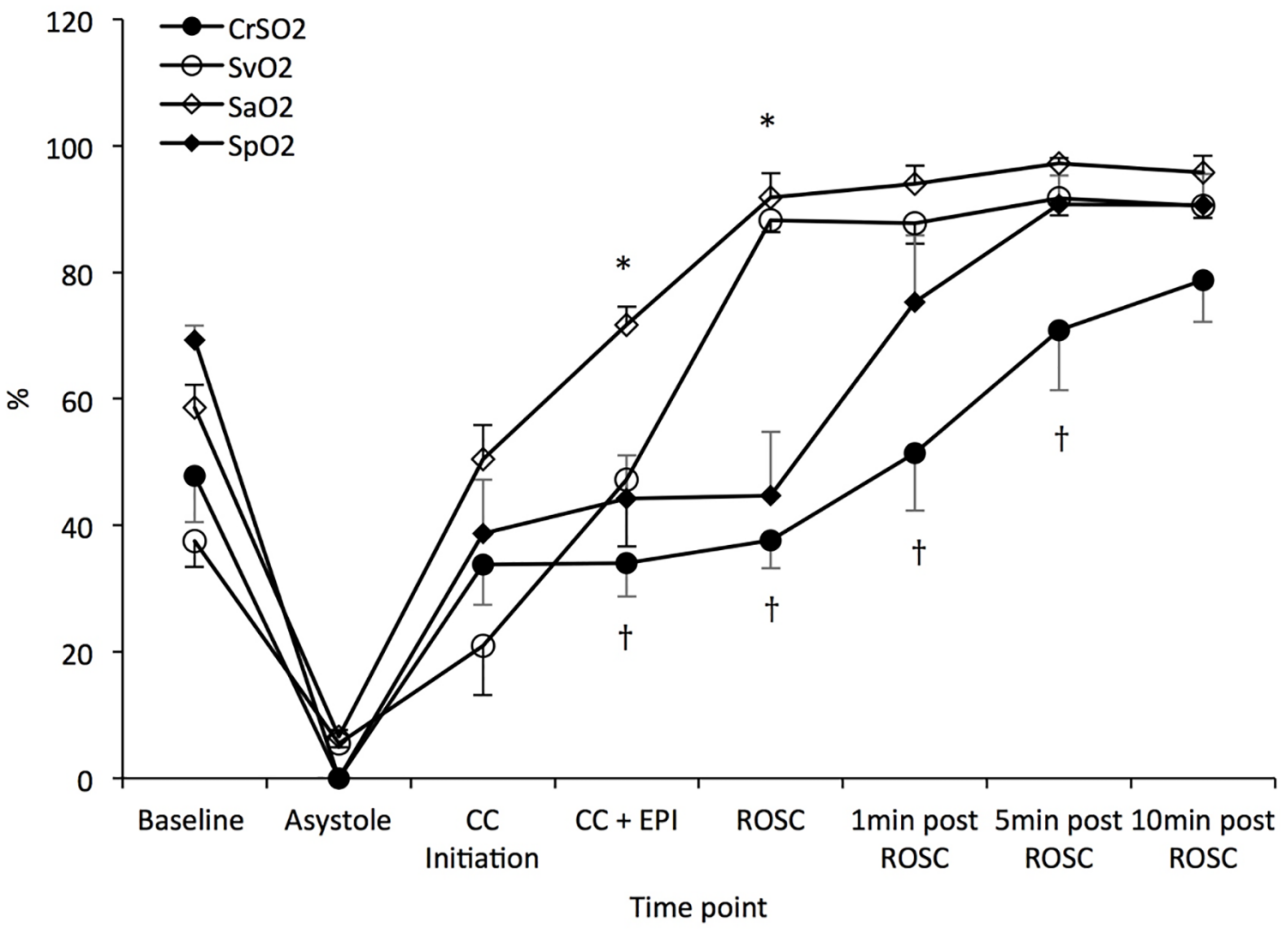
Cerebral rSO_2_, preductal SpO_2_, SaO_2_ and SvO_2_ during study period in lambs with ROSC. Pulse oximetry and NIRS are not reliable during resuscitation as shown by a discrepancy between SaO_2_ and SpO_2_, and SvO_2_ and cerebral rSO_2_ during chest compressions as well as in the first few minutes into ROSC. Data are mean ± SEM bars. * *P* <0.05 SvO_2_ vs. SpO_2_; † *P* <0.05 SvO2 vs. cerebral rSO_2_.

The oxygen hemoglobin saturation from preductal pulse oximeter (SpO_2_) and cerebral NIRS monitor values are shown in Fig 9. The arterial and venous oxygen saturation values derived from co-oximetry from central arterial and venous blood gases (SaO_2_ and SvO_2_, respectively) matched SpO_2_ and CrSO_2_ at baseline and after achieving ROSC. However, during chest compressions in the absence of ROSC, significant SaO_2_—SpO_2_ and SvO_2_ –CrSO_2_ discrepancy was observed (Fig 9).

## Discussion

The recognition that coronary perfusion can revive the inanimate heart and the role for epinephrine in increasing coronary pressures dates back to research from the late 19 to early 20^th^ century.[12] However, it was not until the 1960’s when epinephrine administration and chest compressions in the resuscitation of patients in cardiac arrest became clinically widespread.[13, 14] The evidence to suggest that higher coronary perfusion pressure (CPP) improves success in achieving ROSC with better neurologic outcomes has been well established in the pediatric and adult literature.[15] Unfortunately, few neonatal studies have reported on hemodynamic parameters during resuscitation and, as such, the CPP threshold and the impact of improved CPP in resuscitating neonates remain unknown, especially in the setting of transitioning physiology. Berg et al. compared different methods of “bystander” (no epinephrine) CPR in a piglet model of asphyxia-induced cardiac arrest and provided hemodynamic parameters during resuscitation.[6] The authors have demonstrated that 7/14 piglets randomized to exclusively receive chest compressions achieved ROSC despite mean CPP as low as 2–3 mm Hg. Similarly, we have demonstrated ROSC at low mean diastolic blood pressures of 11 ±4 mm Hg in a model with neonatal transitioning physiology.

During systole, intracardiac blood vessels are compressed and twisted by the spontaneously contracting heart muscle and blood flow to the left ventricle is at its lowest. Flow resumes during diastole when the muscle relaxes.[16] CPP acts as a surrogate for myocardial flow, mathematically calculated as the difference between the arterial diastolic pressure and the right atrial pressure. However, in the arrested heart, when the muscles are not contracting, it is not known whether myocardial flow is greatest during compression (systole) or relaxation (diastole). In addition, the presence of a large ductus arteriosus may prevent a sustained increase in diastolic blood pressure during the recoil phase of chest compressions (due to negative intrathoracic pressure and low pulmonary vascular resistance at arrest). This begets the question whether systolic blood pressures may play a more crucial role in providing forward coronary flow to the newborn’s heart and carotid flow to the brain during compressions. Although the importance in optimizing CPP cannot be dismissed, the threshold CPP that predicts improved survival in neonates remains unknown.

Though we observed an appreciable increase in the mean systolic and diastolic blood pressures (Fig 2), as well as in the maximum left carotid and pulmonary arterial flows (Figs 3 and 4) upon initiation of chest compressions, our data does not suggest a significant rise in blood pressures following the administration of epinephrine as has been previously reported in the literature.[7, 17, 18] There was, also, no significant difference in systolic or diastolic blood pressures, and left carotid or pulmonary arterial flows between the lambs that achieved ROSC and those that did not (Figs 2–6). The lack of hemodynamic response following epinephrine administration may be explained by the severe acidotic state of the animals, as has been previously described.[19] We speculate that the effectiveness of epinephrine in the severely asphyxiated asystolic neonate may be related to its direct β-adrenergic effects on the heart.[20]

These findings are in contrast to a recent study by Sobotka et al. who have reported on the hemodynamic effects of chest compressions in six asphyxiated near-term lambs, wherein the investigators observed that diastolic blood pressures and carotid blood flow did not improve by means of chest compressions alone, unless epinephrine was administered.[7] In establishing our model, we noted that lambs readily achieved ROSC with PPV within seconds of starting chest compressions when the time in asystole was limited to 0–2 minutes. We, therefore, elected to observe a 5-minute period of asystole prior to initiating resuscitation. It is worthwhile mentioning that the lambs in Sobotka et al.’s study approached cardiac arrest despite being ventilated and that resuscitation was begun before absolute circulatory arrest. Some other noteworthy differences in the resuscitation of the lambs in their study may explain the discrepancies among our observations: (1) the heart rate prior to the onset of chest compressions was 72 ±7/min, (2) chest compressions were initiated at a median of 227.5 seconds (range 102 − 344s) from the onset of ventilation, and (3) chest compressions were uninterrupted at a 2.2:1 ratio with ventilation (different from the NRP recommended 3:1 C:V ratio with interruption of chest compressions during ventilation[21]).

Corroborating the findings of the aforementioned study, we also observed reversal of carotid blood flow during the relaxation phase of chest compressions (Fig 6). We, also, demonstrated a similar pattern in the left pulmonary artery, where significant retrograde flow occured during the recoil phase of chest compressions (Fig 5). It has been suggested that the near loss of diastolic blood pressure reduces the critical blood volume within the large arteries below that required to maintain tension on the arterial wall resulting in retrograde blood flow.[7] Furthermore, we speculate that in the transitioning neonatal circulation, shunting of blood across the PDA owing to negative intrathoracic pressures during the relaxation phase of chest compressions limit anterograde flow to periods of chest compression. Improved vascular tone and cardiac output following ROSC establishes exclusive anterograde flow in both the carotid as well as the pulmonary circulations during the systolic and diastolic phases of the cardiac cycle (Figs 5 and 6).

Low PaO_2_ levels achieved during chest compressions in spite of ventilation with 100% oxygen are probably a reflection of the low pulmonary blood flow. So, low arterial oxygen content and low carotid blood flow severely limits oxygen delivery to the brain during chest compressions. This finding indirectly supports the current recommendation to provide 100% oxygen during chest compressions during neonatal resuscitation.[11, 21] However, it is important to immediately decrease FIO_2_ (down to 0.21) as soon as ROSC is achieved to prevent hyperoxia-induced reperfusion injury[22] because gradually decreasing the FIO_2_ maintained PaO_2_ levels >200 mm Hg following the first few minutes after ROSC was established (Fig 8A). Thereafter, the FIO_2_ can be adjusted to achieve the desired oxygen haemoglobin saturation target. In addition, the significant difference between SaO_2_ and SpO_2_, as well as SvO_2_ and cerebral rSO_2_ during resuscitation and the few minutes following ROSC make pulse oximetry and NIRS less reliable during resuscitations requiring chest compressions (Fig 9).

Decreased oxygen consumption and oxygen extraction within minutes of an asphyxial insult has been demonstrated in an experiment of asphyxiated newborn lambs.[23] In a subsequent experiment by the same group assessing mitochondrial function after asphyxia, the authors have reported that in asphyxiated lambs <3 days old, there was a significant depression in mitochondrial respiration as compared to the control group.[24] These findings suggest that asphyxia can impact cellular metabolism with decreased O_2_ extraction/utilization and consequently decreased CO_2_ production. Our blood gas analysis data support these findings: the lambs that did not achieve ROSC demonstrate greater lactic acidosis, suggesting a more pronounced level of hypoxia and tissue compromise. In addition, the lambs that did not achieve ROSC have higher PaO_2_ and lower PaCO_2_ indicating a state of irreversible cellular/mitochondrial dysfunction leading to an increase in circulating PaO_2_ (Fig 8A–8D). The decreasing PaCO_2_ values can be explained as a result of decreased cellular metabolism and elimination through ventilation. Thus, an important factor in the successful resuscitation of the severely asphyxiated neonate may depend on the extent of homeostatic compromise sustained as a result of prolonged tissue hypoperfusion preceding the cardiac arrest. Recent studies in hypoxic newborn piglets have demonstrated that severe hypoxia induces changes in specific metabolites in the plasma metabolome and that using a plasma metabolite score may improve predicting the severity of hypoxic insults, which could clinically help stratify patient treatment.[25, 26] Future experiments involving hypoxic animals should continue to study the plasma metabolome to gain further knowledge regarding the cellular alterations that occur during hypoxia to better target specific therapeutic interventions.

We acknowledge several limitations to this study. A major shortcoming is the significantly smaller number of animals that did not achieved ROSC, and, therefore, the results need to be interpreted carefully. A flow probe was not placed around the ductus arteriosus, limiting our interpretation and understanding of the hemodynamics of chest compression during resuscitation in a model with transitioning circulation. We did not monitor pulmonary arterial pressures due to concerns for catheter dislodgement during chest compressions. Instrumentation of the fetal lambs was performed just prior to our study and may have induced stress. Our model represents asystolic newly born infants. Newborn infants with severe bradycardia requiring chest compressions may demonstrate different hemodynamic responses to chest compressions and epinephrine. Finally, we cannot be certain that some lambs may achieve ROSC if the time to first epinephrine administration is extended.

The use of a neonatal lamb model with transitioning physiology that requires sustained chest compressions to achieve ROSC is a considerable strength of this study. The hemodynamic data collected during chest compressions in this model will generate new knowledge on what parameters may constitute effective chest compressions. End-tidal CO_2_ (ETCO_2_) measurements were collected to assess any correlation between ETCO_2_ and hemodynamic parameters.[27] To our knowledge this is the first study to report on systemic and pulmonary hemodynamic parameters and gas exchange in an asphyxiated model mimicking newborn infants in need for aggressive resuscitation strictly adhering to the current NRP guidelines.[21]

## Conclusion

In this novel term model of asphyxia-induced cardiac arrest with transitioning fetal circulation, mean diastolic blood pressures ≤11 mm Hg are adequate to achieve ROSC. Furthermore, despite resuscitation with 100% oxygen during chest compressions, a low oxygen tension and oxygen content (mean 18 mm Hg and 8 mL O_2_/dL, respectively) seem sufficient to revive the heart. We speculate that independent of chest compression efficiency, an important factor in the successful resuscitation of the asphyxiated neonate depends on the extent of cellular and mitochondrial dysfunction sustained. Further studies focusing on protection against or reversal of cellular dysfunction during neonatal resuscitation are warranted.

## Supporting information

S1 Data Minimal data set. (XLSX)
